# Supplementary material for: A Likelihood Approach for Real-Time Calibration of Stochastic Compartmental Epidemic Models
Source: PLoS Comput Biol. 2017 Jan 17;13(1):e1005257. doi: 10.1371/journal.pcbi.1005257 (PMC5240920; doi:10.1371/journal.pcbi.1005257)
Supplement: S1 File — (TAR.GZ) [file pcbi.1005257.s014.tar.gz › HSPH_Online-SI-Revision/EnKF07/n1-mild/n1-mild_EnKF_table-4.pdf]

1	R0	{1.27677, 7.89375}	{2.46302, 6.4797}	{{1.62638, 4.00995}, {3., 8.74562}}	{96.7969, 32.6753}	inf	17.	54.9936	{8.9461, 81.3952}	245.796	0.381737	newly	24.	56.65	{6., 129.}	186.292	0.386709	Reff	1.16697	0.509787	{0.081789, 0.730393}	63.3515	ar	400.	1825.26	{1656., 2307.}	357.29
2	R0	{1.33231, 10.5259}	{2.44918, 6.98587}	{{0.927559, 3.39631}, {3., 14.7234}}	{98.0169, 50.1932}	inf	39.	49.5648	{3.0184, 77.1835}	120.535	0.497241	newly	28.	51.34	{2., 97.}	165.786	0.505641	Reff	0.927288	0.490624	{0.0919405, 0.847317}	55.7539	ar	467.	1755.62	{1599., 2008.}	275.936
3	R0	{1.2273, 6.76944}	{2.62411, 7.17386}	{{2.15502, 2.62447}, {3., 12.5687}}	{117.499, 34.8576}	inf	19.	207.879	{75.5587, 305.814}	996.157	0.958138	newly	22.	201.79	{74., 372.}	819.773	0.851253	Reff	1.11071	0.811287	{0.243617, 1.54848}	44.8573	ar	314.	1552.99	{1158., 2060.}	394.583
4	R0	{1.41215, 17.5742}	{2.25987, 7.31381}	{{0.702051, 0.414764}, {3., 16.6458}}	{86.9319, 58.4408}	inf	31.	64.3649	{7.99553, 138.842}	150.918	0.351794	newly	15.	58.45	{6., 79.}	315.267	0.436182	Reff	1.27376	0.42719	{0.114744, 0.611008}	70.08	ar	465.	1806.82	{1645., 2087.}	288.563
5	R0	{1.42222, 6.15886}	{2.607, 5.60379}	{{2.14746, 0.834813}, {3., 9.86831}}	{84.6829, 44.8245}	inf	27.	53.8694	{12.9256, 103.746}	118.679	0.28784	newly	31.	88.47	{13., 185.}	217.387	0.341538	Reff	1.21173	0.594373	{0.141369, 1.24395}	65.5093	ar	490.	1768.83	{1583., 2233.}	261.961
6	R0	{1.24363, 9.95596}	{2.59986, 7.02558}	{{2.16013, 2.5211}, {3., 12.902}}	{113.212, 40.4914}	inf	12.	207.312	{68.7027, 374.217}	1628.84	1.15242	newly	7.	219.78	{66., 497.}	3039.71	1.35085	Reff	1.21503	0.8487	{0.235154, 1.60611}	48.8099	ar	411.	1559.67	{1066., 2185.}	279.774
7	R0	{1.36478, 5.96056}	{2.54098, 7.49518}	{{1.86811, 2.48509}, {3., 13.4107}}	{86.8383, 50.5764}	inf	11.	189.225	{77.5909, 304.246}	1622.3	1.14037	newly	13.	170.24	{38., 290.}	1212.62	0.991803	Reff	1.2092	0.614879	{0.189537, 1.04141}	58.7711	ar	484.	1571.85	{1086., 2217.}	224.762
8	R0	{1.36335, 17.6429}	{2.4245, 5.16722}	{{0.885116, 1.22917}, {3., 10.1318}}	{94.5748, 70.7122}	inf	30.	33.9929	{3.7693, 28.308}	93.3586	0.38774	newly	14.	42.84	{3., 38.}	251.857	0.415684	Reff	1.27473	0.453545	{0.107798, 0.839924}	65.8175	ar	484.	1683.47	{1613., 1752.}	247.824
9	R0	{1.26575, 5.28707}	{2.79554, 7.3062}	{{2.41985, 3.62076}, {3., 9.64123}}	{120.86, 56.4969}	inf	13.	52.7409	{5.66675, 125.04}	329.285	0.478507	newly	20.	65.14	{4., 175.}	274.7	0.43334	Reff	1.05943	0.611576	{0.0858175, 1.56011}	67.3543	ar	472.	1813.93	{1620., 2410.}	284.307
10	R0	{1.42942, 9.49905}	{2.43626, 4.60926}	{{0.935438, 0.0974172}, {3., 9.05498}}	{87.1887, 53.3381}	inf	45.	53.279	{8.41257, 70.9294}	102.512	0.397295	newly	34.	63.56	{8., 192.}	148.529	0.368703	Reff	1.07635	0.481484	{0.125708, 1.01251}	61.7815	ar	468.	1774.64	{1626., 2027.}	279.197
11	R0	{1.46827, 15.0983}	{2.81174, 6.80413}	{{2.42382, 3.61651}, {3., 18.277}}	{91.4999, 60.799}	inf	28.	41.1726	{3.15663, 40.3608}	117.005	0.389618	newly	14.	62.08	{2., 106.}	389.143	0.462262	Reff	1.36843	0.520544	{0.112198, 1.19709}	70.5261	ar	495.	1758.12	{1581., 2434.}	255.176
12	R0	{1.25751, 13.279}	{2.42397, 6.1951}	{{0.948029, 0.507182}, {3., 10.4587}}	{104.886, 54.9373}	inf	18.	41.0591	{6.82632, 42.2041}	155.778	0.279128	newly	8.	50.82	{6., 79.}	549.25	0.498706	Reff	1.17829	0.455312	{0.0745976, 0.682305}	66.1816	ar	366.	1718.11	{1629., 1779.}	369.429
13	R0	{1.3425, 9.16361}	{2.61464, 5.53777}	{{1.73984, 1.08325}, {3., 7.97896}}	{99.2832, 41.1757}	inf	32.	42.3534	{9.17489, 82.5731}	92.686	0.337003	newly	28.	62.6	{8., 138.}	187.143	0.434881	Reff	0.965258	0.534707	{0.137478, 0.998721}	58.8773	ar	442.	1768.35	{1617., 2173.}	300.079
14	R0	{1.18936, 6.20583}	{2.52022, 5.74583}	{{2.15167, 0.692568}, {3., 10.002}}	{114.09, 42.3113}	inf	26.	64.1963	{10.4377, 148.556}	170.481	0.349789	newly	32.	114.08	{9., 272.}	294.5	0.402277	Reff	1.01333	0.692558	{0.113862, 2.14952}	69.5998	ar	382.	1824.24	{1587., 2537.}	378.351
15	R0	{1.27137, 9.78868}	{2.55344, 5.72844}	{{2.1467, 0.88463}, {3., 10.1058}}	{102.326, 45.1437}	inf	27.	66.0695	{12.1525, 129.807}	165.204	0.337063	newly	24.	109.3	{11., 256.}	378.583	0.430091	Reff	1.10101	0.659709	{0.139877, 2.14456}	67.0383	ar	496.	1820.3	{1586., 2529.}	267.589
16	R0	{1.23102, 13.6452}	{2.26016, 5.02662}	{{0.672339, 1.28903}, {3., 11.3207}}	{106.911, 63.162}	inf	33.	63.2771	{5.98611, 125.478}	151.993	0.431155	newly	16.	75.83	{5., 89.}	404.188	0.501262	Reff	1.07222	0.419204	{0.111, 0.664158}	66.5833	ar	445.	1798.91	{1641., 1919.}	304.249
17	R0	{1.27213, 13.7452}	{2.07033, 4.92103}	{{0.191754, 1.90424}, {3., 7.62807}}	{102.367, 65.8264}	inf	22.	101.338	{10.7478, 256.739}	380.4	0.558941	newly	11.	108.	{9., 251.}	892.909	0.787494	Reff	1.19962	0.382948	{0.0347726, 0.580774}	69.1257	ar	367.	1628.22	{1373., 1932.}	343.657
18	R0	{1.25318, 11.2151}	{2.83964, 6.54456}	{{2.45197, 0.688114}, {3., 17.6573}}	{126.595, 57.5593}	inf	26.	36.1215	{4.84653, 30.8677}	108.984	0.336631	newly	13.	47.	{4., 39.}	302.154	0.368009	Reff	1.10029	0.452981	{0.171649, 0.678067}	65.1325	ar	416.	1726.	{1591., 1941.}	314.904
19	R0	{1.46006, 8.11496}	{2.08318, 4.69697}	{{0.171291, 1.80504}, {3., 5.75538}}	{81.7051, 49.5716}	inf	42.	97.784	{10.3586, 239.15}	167.066	0.401035	newly	40.	108.63	{13., 290.}	206.275	0.41912	Reff	1.24251	0.39014	{0.030229, 0.608515}	70.2282	ar	496.	1614.04	{857., 2091.}	227.129
20	R0	{1.14273, 6.71261}	{2.25018, 5.17168}	{{0.677145, 2.02476}, {3., 11.5973}}	{118.422, 54.7658}	inf	15.	69.1376	{6.00176, 151.048}	387.881	0.457905	newly	12.	73.81	{6., 114.}	536.75	0.494551	Reff	1.02046	0.424215	{0.101007, 0.702771}	67.324	ar	362.	1850.64	{1666., 2103.}	411.227
21	R0	{1.42432, 8.23905}	{2.31743, 3.29773}	{{0.783804, 0.463012}, {3., 7.60907}}	{85.2428, 59.9744}	inf	17.	75.7891	{6.8659, 176.633}	367.624	0.497085	newly	14.	101.51	{10., 265.}	639.071	0.585216	Reff	1.15512	0.413328	{0.126322, 0.619324}	69.0643	ar	405.	1838.62	{1639., 2151.}	353.98
22	R0	{1.29386, 17.863}	{2.54872, 5.33942}	{{2.13643, 0.504544}, {3., 9.98866}}	{98.7944, 70.109}	inf	27.	49.1809	{12.896, 90.994}	103.707	0.282395	newly	8.	94.11	{10., 161.}	1083.13	0.72825	Reff	1.20588	0.588965	{0.111142, 1.52099}	67.5931	ar	488.	1818.34	{1638., 2357.}	272.611
23	R0	{1.39274, 13.0167}	{2.40517, 7.18401}	{{0.944568, 4.43768}, {3., 13.4696}}	{88.7314, 49.8181}	inf	34.	39.5739	{3.2867, 42.2878}	110.131	0.472951	newly	12.	39.62	{2., 58.}	274.833	0.443031	Reff	1.12533	0.502132	{0.0931857, 0.954129}	64.199	ar	475.	1738.77	{1595., 1966.}	266.057
24	R0	{1.32178, 15.8991}	{2.63776, 7.7655}	{{2.26413, 0.252505}, {3., 17.003}}	{104.745, 54.3721}	inf	43.	42.1831	{4.50727, 49.2701}	83.8256	0.421728	newly	19.	63.37	{7., 57.}	281.526	0.376286	Reff	1.16978	0.536081	{0.134423, 0.876347}	60.6367	ar	471.	1776.76	{1640., 1962.}	277.231
25	R0	{1.37568, 17.8427}	{2.7932, 7.69438}	{{2.43462, 3.60495}, {3., 13.2478}}	{103.041, 56.8766}	inf	18.	30.4078	{15.7758, 28.263}	87.2398	0.150353	newly	6.	33.84	{6., 29.}	469.333	0.440125	Reff	1.20647	0.575083	{0.221684, 1.09499}	62.9186	ar	398.	1692.33	{1569., 2040.}	325.209
26	R0	{1.20993, 6.4656}	{2.62807, 7.74544}	{{1.20801, 0.244404}, {3., 16.2387}}	{124.418, 73.6345}	inf	13.	41.3589	{2.30299, 50.4985}	248.434	0.469738	newly	16.	62.62	{1., 76.}	337.375	0.503527	Reff	1.03933	0.543441	{0.152001, 0.860913}	58.6484	ar	345.	1808.38	{1645., 2108.}	424.168
27	R0	{1.20548, 13.8003}	{2.80344, 7.28111}	{{2.41998, 3.62062}, {3., 14.8695}}	{132.558, 51.4298}	inf	42.	25.5264	{8.25648, 24.0689}	72.1691	0.437879	newly	24.	30.37	{5., 26.}	111.375	0.392646	Reff	1.0548	0.428787	{0.0854691, 0.639818}	68.3957	ar	350.	1690.26	{1574., 1909.}	382.931
28	R0	{1.3176, 14.6594}	{2.52057, 4.96728}	{{2.12802, 0.381668}, {3., 7.01463}}	{94.5294, 66.2894}	inf	14.	51.386	{13.5112, 102.176}	275.9	0.492067	newly	4.	105.98	{13., 183.}	2553.	1.05776	Reff	1.27412	0.607184	{0.138587, 1.54754}	66.9297	ar	362.	1854.75	{1652., 2457.}	412.362
29	R0	{1.28202, 6.23047}	{2.61867, 7.99213}	{{1.30852, 0.929313}, {3., 16.0628}}	{111.115, 67.6021}	inf	36.	41.9075	{3.99392, 61.1878}	95.6118	0.402964	newly	35.	61.81	{3., 98.}	181.457	0.588749	Reff	1.10382	0.575037	{0.124277, 1.03531}	58.2914	ar	391.	1820.49	{1648., 2083.}	365.598
30	R0	{1.28177, 8.83252}	{2.04652, 5.34254}	{{0.23002, 1.76455}, {3., 7.33266}}	{98.8034, 50.5882}	inf	30.	96.1505	{10.033, 156.342}	246.572	0.466627	newly	21.	95.72	{9., 155.}	375.333	0.545269	Reff	1.12539	0.387524	{0.0281193, 0.629282}	67.3093	ar	427.	1599.54	{625., 2006.}	276.974
31	R0	{1.35886, 5.97708}	{2.5271, 7.13661}	{{1.62291, 3.78}, {3., 12.0128}}	{86.5226, 45.0452}	inf	23.	54.7503	{5.74374, 81.0071}	174.981	0.378492	newly	44.	62.87	{3., 94.}	136.977	0.559459	Reff	1.14008	0.481879	{0.06036, 0.753669}	63.8714	ar	491.	1828.84	{1643., 2304.}	272.473
32	R0	{1.27601, 17.9804}	{2.58984, 9.39752}	{{2.24733, 0.91095}, {3., 17.294}}	{107.184, 47.8967}	inf	25.	38.1601	{3.47717, 46.1454}	97.4312	0.298805	newly	8.	57.99	{3., 29.}	650.625	0.440456	Reff	1.18924	0.567237	{0.102851, 1.0313}	59.4542	ar	445.	1767.06	{1634., 1882.}	297.092
33	R0	{1.3368, 11.3235}	{2.26474, 4.66697}	{{0.690016, 2.04796}, {3., 10.1211}}	{94.0799, 59.0211}	inf	40.	59.9056	{4.84755, 102.253}	123.649	0.411158	newly	21.	67.11	{5., 81.}	257.286	0.382907	Reff	1.1697	0.407962	{0.102797, 0.660886}	69.9994	ar	417.	1830.78	{1672., 1962.}	339.036
34	R0	{1.21021, 11.3635}	{2.59371, 8.23908}	{{2.2823, 0.453139}, {3., 17.9477}}	{118.572, 56.1892}	inf	20.	47.6389	{1.68117, 60.208}	180.855	0.408811	newly	14.	82.16	{1., 100.}	535.	0.528719	Reff	1.06862	0.62492	{0.162338, 1.19653}	58.9589	ar	327.	1836.77	{1652., 2553.}	461.703
35	R0	{1.53382, 10.8866}	{2.44862, 6.95362}	{{1.61773, 3.96927}, {3., 15.1795}}	{63.6477, 46.7892}	inf	30.	57.2648	{5.53361, 75.9355}	130.979	0.323208	newly	22.	57.85	{4., 111.}	211.227	0.415304	Reff	1.3789	0.48509	{0.034066, 0.762717}	68.4727	ar	476.	1863.56	{1650., 2313.}	291.504
36	R0	{1.20423, 6.83844}	{1.95184, 5.1729}	{{0.179687, 1.60435}, {3., 6.3063}}	{105.734, 38.9291}	inf	44.	104.372	{14.8307, 217.772}	169.898	0.384712	newly	49.	103.93	{13., 268.}	154.714	0.382659	Reff	1.05852	0.364122	{0.0279692, 0.605773}	66.5038	ar	416.	1639.12	{1403., 2002.}	294.091
37	R0	{1.43629, 10.1548}	{2.66227, 7.33971}	{{2.27463, 0.445414}, {3., 17.4597}}	{89.5668, 62.7157}	inf	15.	53.2884	{3.52034, 49.8522}	286.849	0.449789	newly	13.	87.67	{2., 69.}	609.769	0.504425	Reff	1.27255	0.576341	{0.133317, 0.914497}	62.887	ar	367.	1827.23	{1642., 2360.}	397.883
38	R0	{1.37504, 8.93428}	{2.04698, 5.25787}	{{0.199761, 1.71364}, {3., 7.34284}}	{88.8361, 51.3333}	inf	32.	96.3098	{10.1553, 155.331}	226.908	0.442036	newly	24.	95.09	{10., 153.}	319.708	0.492662	Reff	1.17703	0.388174	{0.0317896, 0.635197}	67.4617	ar	483.	1597.	{838., 2001.}	231.971
39	R0	{1.51637, 9.36742}	{2.51084, 7.50716}	{{1.65506, 2.66084}, {3., 13.7153}}	{68.6547, 50.1809}	inf	43.	56.9508	{7.10193, 120.533}	97.6629	0.380913	newly	31.	71.47	{4., 102.}	195.065	0.465391	Reff	1.204	0.54147	{0.100847, 0.734359}	62.0852	ar	441.	1853.24	{1655., 2399.}	320.236
40	R0	{1.23658, 9.82869}	{2.23903, 7.04481}	{{1.2117, 4.12135}, {3., 13.5554}}	{89.5311, 47.3297}	inf	29.	136.24	{28.6579, 261.327}	379.281	0.556089	newly	15.	119.73	{17., 235.}	704.467	0.709508	Reff	1.09561	0.494095	{0.114763, 1.05953}	65.4657	ar	414.	1595.41	{1185., 1953.}	285.365
41	R0	{1.24247, 5.4242}	{2.55813, 7.15385}	{{1.87489, 2.07612}, {3., 13.1998}}	{106.871, 59.3581}	inf	23.	185.87	{74.6543, 327.333}	712.82	0.816827	newly	36.	174.07	{40., 357.}	390.583	0.56992	Reff	1.1381	0.621825	{0.133724, 1.10138}	56.6768	ar	345.	1559.46	{1090., 2097.}	352.017
42	R0	{1.28208, 4.40223}	{2.2395, 7.12184}	{{0.85772, 3.87831}, {3., 13.4676}}	{84.4121, 69.0743}	inf	20.	149.205	{27.812, 353.626}	653.355	0.723337	newly	30.	133.82	{22., 338.}	361.	0.521607	Reff	1.1949	0.540565	{0.120057, 1.28223}	62.7319	ar	339.	1618.33	{1173., 2099.}	377.383
43	R0	{1.56773, 6.50474}	{2.56853, 6.34129}	{{2.14664, 2.60113}, {3., 12.3353}}	{70.528, 39.3436}	inf	33.	225.634	{68.1655, 404.123}	587.852	0.754423	newly	44.	259.5	{73., 667.}	495.091	0.644742	Reff	1.34982	0.856236	{0.206152, 1.94928}	51.1508	ar	498.	1533.5	{962., 2177.}	208.297
44	R0	{1.10489, 6.01941}	{2.63388, 6.10414}	{{1.83365, 1.09151}, {3., 9.97369}}	{140.323, 39.6808}	inf	12.	30.1585	{5.54345, 34.2846}	175.675	0.342438	newly	14.	52.1	{5., 64.}	313.714	0.42592	Reff	0.942471	0.527799	{0.0533757, 1.07055}	61.7903	ar	346.	1781.77	{1630., 2196.}	414.962
45	R0	{1.25888, 14.1926}	{2.80787, 5.55318}	{{2.41235, 0.411654}, {3., 7.71697}}	{123.045, 63.0838}	inf	50.	39.1375	{5.35095, 36.0392}	95.623	0.538043	newly	23.	42.31	{4., 43.}	147.783	0.373097	Reff	0.981926	0.484517	{0.256868, 0.62998}	56.0469	ar	471.	1669.34	{1576., 1757.}	254.425
46	R0	{1.45292, 16.616}	{1.95845, 4.85064}	{{0.177063, 1.68343}, {3., 5.84675}}	{82.9883, 71.2118}	inf	34.	99.0299	{12.1761, 266.134}	217.012	0.444898	newly	17.	103.88	{10., 258.}	527.882	0.656117	Reff	1.34105	0.360355	{0.0331302, 0.548696}	73.7681	ar	494.	1652.14	{1497., 1992.}	234.441
47	R0	{1.22528, 16.6033}	{2.41188, 6.51707}	{{1.61491, 4.16623}, {3., 8.80312}}	{98.244, 60.7483}	inf	14.	64.9576	{10.1306, 136.543}	377.792	0.46604	newly	6.	75.03	{8., 135.}	1160.5	0.751943	Reff	1.17014	0.559053	{0.199077, 1.25059}	60.6379	ar	368.	1884.23	{1656., 2424.}	412.644
48	R0	{1.1669, 6.92715}	{2.80109, 4.81639}	{{2.4427, 2.97506}, {3., 6.63272}}	{140.046, 35.2162}	inf	17.	52.9198	{5.1646, 44.7061}	244.79	0.319666	newly	17.	78.	{5., 109.}	395.059	0.415421	Reff	0.977862	0.63881	{0.205896, 1.7476}	66.4126	ar	416.	1786.97	{1597., 2136.}	329.56
49	R0	{1.32749, 16.0887}	{2.17397, 4.37703}	{{0.608273, 1.95383}, {3., 10.5303}}	{93.1328, 72.7944}	inf	24.	52.5671	{5.71346, 61.432}	168.271	0.370812	newly	8.	64.63	{5., 70.}	722.625	0.588385	Reff	1.24917	0.395459	{0.0951742, 0.6754}	72.5896	ar	353.	1804.32	{1647., 1917.}	411.139
50	R0	{1.29636, 15.2609}	{2.49835, 4.5404}	{{0.879087, 0.14609}, {3., 10.1074}}	{105.535, 70.8502}	inf	50.	37.8694	{4.26495, 73.2562}	79.2636	0.50161	newly	20.	79.32	{3., 296.}	347.1	0.526775	Reff	1.03838	0.539399	{0.124682, 0.951814}	59.1138	ar	470.	1726.69	{1594., 2118.}	267.381
